# Rapid and sensitive detection of *Pseudomonas aeruginosa* in traditional Chinese medicine herbal pieces using loop-mediated isothermal amplification combined with fluorescent microsphere–based lateral flow strip

**DOI:** 10.3389/fcimb.2026.1861254

**Published:** 2026-07-22

**Authors:** Wenlin Tian, Chao Yang, Zhilan Chen, Jingtao Wen, Yujie Zhong, Yongcan Guo

**Affiliations:** 1 Nanobiosensing and Microfluidic Point-of-Care Testing, Key Laboratory of Luzhou, Department of Clinical Laboratory, The Affiliated Traditional Chinese Medicine Hospital, Southwest Medical University, Luzhou, Sichuan, China; 2 Laboratory Department, Hejiang County Traditional Chinese Medicine Hospital, Luzhou, Sichuan, China

**Keywords:** ecfX gene, herbal matrix inhibition, lateral flow strip, loop-mediated isothermal amplification, on-site detection, *Pseudomonas aeruginosa*, traditional Chinese medicine

## Abstract

**Objective:**

*Pseudomonas aeruginosa (P. aeruginosa)* is a common contaminant in traditional Chinese medicine (TCM) herbal pieces, and its detection is often hindered by polysaccharides, polyphenols, and other inhibitory compounds in the herbal matrix. This study aimed to develop a rapid, sensitive, and matrix-tolerant method for detecting P. aeruginosa in TCM herbal pieces for routine quality control.

**Methods:**

A loop-mediated isothermal amplification (LAMP) assay targeting the species-specific *ecfX* gene was developed and integrated with a fluorescent microsphere–based lateral flow strip (LFS) for visual result interpretation. LAMP primers were dual labeled with digoxigenin and biotin to enable specific amplicon to capture at the test and control lines of the LFS. Reaction conditions for both LAMP reaction and LFS were optimized using genomic DNA extracted from *P. aeruginosa*-spiked powdered herbal samples. Analytical performance, including specificity, sensitivity, reproducibility, and limit of detection (LOD), was rigorously evaluated. The validated method was then applied to six commonly used TCM herbal pieces, including Astragali radix and Angelicae sinensis radix. Additionally, the assay performance was validated using six representative TCM herbal pieces and 90 clinical-relevant herbal samples in parallel with qPCR.

**Results:**

The LAMP-LFS assay demonstrated strict specificity for *P. aeruginosa*, with no cross-reactivity observed against any non-target bacterial strains tested. Optimal LAMP conditions were determined as 65 °C for 60 min in the presence of 6 mM Mg^2+^ and 1.4 mM dNTPs. The entire detection workflow, including DNA extraction, amplification, and strip readout, was completed within approximately 70 min. The assay achieved a limit of detection of 1 × 10² CFU/250 mL in spiked herbal matrices, with high reproducibility (CV < 5%). Consistent detection was observed across six different TCM herbal species, indicating strong tolerance to complex phytochemical backgrounds. In parallel validation with qPCR, the assay demonstrated a sensitivity of 94.0%, specificity of 97.5%, and overall accuracy of 95.6%, with a Cohen’s kappa coefficient of 0.92.

**Conclusion:**

The developed fluorescent microsphere–based LAMP-LFS assay provides a rapid, sensitive, and reproducible platform for detecting *P. aeruginosa* in TCM herbal pieces. Compared with conventional PCR-based approaches, this method demonstrates improved tolerance to inhibitory herbal matrices and enables reliable on-site applicability with minimal instrumentation requirements. The assay shows potential for routine microbiological quality control and field-based monitoring of TCM herbal materials.

## Introduction

*Pseudomonas aeruginosa (P. aeruginosa)* is an opportunistic Gram-negative bacterium ubiquitous in water systems and moist environments, and a well-recognized microbial contaminant of non-sterile pharmaceutical and herbal preparations (Gholipour et al., 2024). Its intrinsic resistance to a broad spectrum of environmental stresses, disinfectants, and antimicrobial agents enables survival in liquid formulations, medical devices, and water-based products (Wang et al., 2025). Consequently, *P. aeruginosa* can compromise product quality, shorten shelf life, and pose serious health risks, particularly to immunocompromised patients (Roy et al., 2023). Therefore, rapid and reliable detection of *P. aeruginosa* is an essential component of microbiological quality control in traditional Chinese medicine (TCM) herbal materials and related products.

TCM herbal pieces are widely used in clinical practice and are typically produced by cleaning, slicing, and drying raw herbal materials (Zhang et al., 2025; Ren et al., 2026). However, microbial contamination may occur during cultivation, harvesting, transportation, processing, storage, and distribution. In addition, TCM herbal matrices contain abundant polysaccharides, polyphenols, pigments, tannins, essential oils, and other secondary metabolites that can interfere with nucleic acid extraction and amplification (Zhu et al., 2024; Guo et al., 2025; Yang et al., 2025). These matrix-associated inhibitors substantially reduce the performance of conventional molecular assays and make accurate microbial detection in herbal products more challenging than in many food and pharmaceutical matrices.

Loop-mediated isothermal amplification (LAMP) is an isothermal nucleic acid amplification technique that enables rapid and specific detection of target DNA without the need for thermal cycling (Peirano et al., 2024). Compared with conventional PCR, LAMP generally exhibits greater tolerance to amplification inhibitors and can be combined with lateral flow strip (LFS) technology to provide rapid and visual result interpretation (Nwe et al., 2024; Diao et al., 2026). These characteristics make LAMP-LFS an attractive platform for point-of-need pathogen detection in resource-limited settings. Several studies have reported the application of LAMP or LAMP-LFS assays for the detection of *P. aeruginosa* in clinical, environmental, and food samples (Lai et al., 2023; Jang et al., 2024). Among the available molecular targets, the *ecfX* gene has been widely used because of its high species specificity and sequence conservation within *P. aeruginosa* (He et al., 2025). Previous studies have demonstrated satisfactory analytical performance of *ecfX*-targeted LAMP assays and LAMP-LFS platforms in relatively simple sample matrices (Jang et al., 2024). However, their performance in TCM herbal materials has not been extensively evaluated. Therefore, further studies are needed to assess the applicability of LAMP-LFS in complex herbal matrices.

In the present study, we developed a fluorescent microsphere–based LAMP-LFS assay targeting the species-specific *ecfX* gene for the detection of *P. aeruginosa* in TCM herbal pieces. The assay combines dual-labeled LAMP amplification with fluorescent lateral flow strip readout and was optimized using representative herbal matrices. By evaluating its analytical performance in multiple TCM herbal samples, this study aimed to assess the feasibility of a rapid and sensitive detection approach for *P. aeruginosa* in complex herbal matrices and to evaluate its suitability for microbiological quality monitoring of TCM herbal materials.

## Materials and methods

### Reagents and instruments

Bst 3.0 DNA polymerase, 10× Thermopol reaction buffer, dNTP mixture, and MgSO_4_ were purchased from New England Biolabs (NEB, USA). EvaGreen Dye (20×) was obtained from Biotium (USA). Tween-20 and Triton X-100 were purchased from Amresco (USA). Streptavidin (SA) was obtained from Solarbio (China). Carboxyl-functionalized fluorescent microspheres (FMs) were purchased from Merck Millipore (USA). 2-(N-morpholino) ethanesulfonic acid monohydrate (MES), N-hydroxysulfosuccinimide (Sulfo-NHS), and 1-ethyl-3-(3-dimethylaminopropyl) carbodiimide hydrochloride (EDC) were obtained from Sangon Biotech (Shanghai, China). All other reagents were of analytical grade and prepared using nuclease-free water. A fluorescence quantitative PCR instrument was purchased from Tianlong Technology (Xi’an, China). An isothermal amplification instrument was purchased from Biometra (Germany). A programmable dispenser for strip preparation was purchased from Jiening Biotechnology (Shanghai, China). A Zetasizer Nano ZS (Malvern Panalytical, UK) was used for dynamic light scattering (DLS) and Zeta potential measurements.

### Target gene selection and LAMP primer design

The *ecfX* gene, a species-specific and highly conserved gene of *P. aeruginosa*, was selected as the target for LAMP assay development (He et al., 2025). Genomic DNA of *P. aeruginosa* (ATCC 27853) and non-target bacterial strains, including Escherichia coli (ATCC 25922), Staphylococcus aureus (ATCC 29213), and Bacillus subtilis (ATCC 6051), Pseudomonas fluorescens (ATCC 13525), Pseudomonas putida (ATCC 12633), and Bacillus cereus (ATCC 14579) was extracted using a commercial bacterial genomic DNA extraction kit according to the manufacturer’s instructions. For spiked TCM herbal piece samples, additional grinding and lysis steps were performed to ensure efficient recovery of bacterial DNA.

LAMP primers were designed based on the conserved region of the *ecfX* gene using PrimerExplorer V5 (Eiken Chemical Co., Japan). The primer set consisted of two outer primers (F3 and B3) and two inner primers (FIP and BIP). To facilitate downstream detection using a lateral flow strip, FIP and BIP primers were 5′-labeled with digoxigenin (Dig) and biotin, respectively, allowing dual-labeled amplicons to be captured on the test (T) and control (C) lines. All primers were synthesized by Sangon Biotech (Shanghai, China). Sequences are listed in **Table 1**.

**Table 1:** Primers used for the LAMP assay targeting the *ecfX* gene of *P. aeruginosa*.

Primer name	Sequence (5′→3′)	Length	Modification	Function
F3	CCGTTTCCGCGTGGTTTC	18 nt	None	Outer forward primer
B3	CGCCAGCGTCAGGTAGTC	18 nt	None	Outer backward primer
FIP	F1c: ACCAGCGTGACTGGTACGGT F2: TGCTGGTCTTCGACCAGAC	39 nt	Digoxigenin (5′)	Forward inner primer (F1c + F2)
BIP	B1c: CCAGCCGACCTCAACGAC B2: GATCGCCAGCGTATTGAAG	38 nt	Biotin (5′)	Backward inner primer (B1c + B2)

Assay specificity was evaluated by monitoring fluorescence amplification curves for *P. aeruginosa* and non-target bacterial DNA. All experiments were performed in triplicate to ensure reproducibility. Positive amplification was expected only for *P. aeruginosa*, while non-target bacterial DNA and blank controls should remain negative.

### DNA extraction from TCM herbal piece samples

Genomic DNA of *P. aeruginosa* (ATCC 27853) and non-target bacterial strains was extracted using a commercial bacterial genomic DNA extraction kit (Ezup Column Plant Genomic DNA Purification Kit, Sangon Biotech, Shanghai, China; Cat# B518261) according to the manufacturer’s instructions. For TCM herbal piece samples spiked with *P. aeruginosa*, the following modifications were implemented to overcome matrix-related inhibition caused by polysaccharides, polyphenols, and pigments: (i) approximately 0.1 g of powdered herbal sample was ground in liquid nitrogen for 5 min to ensure complete homogenization; (ii) the lysis incubation was performed at 65 °C for 30 min with vortex mixing every 10 min; and (iii) the final DNA was eluted in 30 μL of nuclease-free water to maximize DNA concentration. DNA purity and concentration were assessed by measuring the A260/A280 ratio using a NanoDrop spectrophotometer (Thermo Fisher Scientific, USA), and only samples with A260/A280 ratios between 1.8 and 2.0 were used for subsequent LAMP assays.

### LAMP reaction system and optimization of amplification conditions

The LAMP reaction was performed in a total volume of 25 μL containing 8 U of Bst 3.0 DNA polymerase, 2.5 μL of 10× Thermopol reaction buffer, 1.4 mM dNTPs, 6 mM MgSO_4_, 1.25 μL EvaGreen Dye, 1.6 μmol·L⁻¹ each of FIP and BIP primers, 0.2 μmol·L⁻¹ each of F3 and B3 primers, 1 μL of DNA template, and nuclease-free water to volume.

Real-time fluorescence monitoring was conducted using a quantitative PCR instrument under isothermal conditions. To optimize LAMP reaction conditions for herbal piece matrices, spiked herbal piece DNA samples were tested under temperature gradients (60–67 °C), Mg²^+^ concentrations (4–8 mM), dNTP concentrations (1.0–1.6 mM), and reaction times (30–70 min). Amplification products were analyzed by fluorescence curves, and the conditions producing the fastest amplification with clear ladder-like bands were selected as optimal.

### Preparation and characterization of fluorescent microsphere–streptavidin (FMs-SA) conjugates

FMs-SA conjugates were prepared using a two-step covalent coupling method based on EDC/Sulfo-NHS chemistry. Briefly, 50 μL of carboxyl-functionalized fluorescent microspheres (2 mg·mL⁻¹) were mixed with 450 μL MES buffer and centrifuged at 12,000 r·min⁻¹ for 15 min. After removal of the supernatant, the microspheres were washed twice with MES buffer and resuspended. EDC (19.2 mg) and Sulfo-NHS (21.7 mg) were dissolved in 2 mL ultrapure water, and 12 μL of the mixture was added to the microsphere suspension. The mixture was incubated with gentle rotation for 20 min to activate carboxyl groups on the microsphere surface. Subsequently, 16 μL of streptavidin solution (1 mg·mL⁻¹) was added, followed by incubation with rotation for 3 h. After centrifugation, the conjugates were blocked overnight at 4 °C in Tris-HCl buffer. Finally, the FMs-SA conjugates were resuspended in Tris-HCl buffer containing 5% (w/v) sucrose and stored at 4 °C in the dark. Successful conjugation was confirmed using fluorescence spectroscopy and UV–visible absorption spectroscopy. Successful conjugation was confirmed using fluorescence spectroscopy and UV–visible absorption spectroscopy, with criteria including appearance of a strong emission peak at 532 nm, UV absorption at 280 nm, and absence of visible aggregation. To further assess colloidal stability and conjugation efficiency, hydrodynamic diameter and polydispersity index (PDI) were measured by DLS, and surface charge was determined by Zeta potential analysis using a Zetasizer Nano ZS (Malvern Panalytical, UK). Briefly, bare FMs and FMs-SA conjugates were diluted 1:100 (v/v) in 10 mM phosphate-buffered saline (pH 7.4) to avoid multiple scattering effects. All measurements were performed in triplicate at 25 °C. An increase in Z-average diameter and a significant shift in Zeta potential were considered indicative of successful conjugation.

### Preparation for lateral flow strips

Lateral flow strips were assembled using standard components, including a sample pad, conjugate pad, nitrocellulose membrane, absorbent pad, and backing card. The prepared FMs-SA conjugates were dispensed onto the conjugate pad using a programmable dispenser and dried at room temperature.

Digoxigenin antibody (anti-Dig) and biotinylated bovine serum albumin (BSA-biotin) were immobilized on the nitrocellulose membrane as the test line (T line) and control line (C line), respectively, and dried prior to assembly.

During detection, dual-labeled LAMP amplicons from herbal piece DNA samples were applied to the strips. Fluorescence signals at the T and C lines were recorded using a fluorescence reader, enabling qualitative and semi-quantitative analysis.

### Optimization of lateral flow strip conditions and limit of detection

Key parameters affecting strip performance, including running buffer type and concentration, FMs–SA loading amount, and development time, were systematically optimized. Running buffers tested included Tris-HCl (0.5% Tween-20), 1× SSC (0.5% Tween-20), 5× SSC (0.5% Tween-20) and PBST (0.5% Tween-20). FMs–SA conjugate volumes (5–20 μL per strip) and strip development times (5–20 min) were compared to identify conditions yielding highest signal-to-noise ratio.

For LOD determination, serial dilutions of *P. aeruginosa ecfX* gene templates (10^1–^10^5^ CFU/250 mL) were spiked into powdered herbal pieces. LAMP amplification was performed under optimized conditions, followed by LFS detection. The lowest concentration consistently producing a visible T line and measurable fluorescence in three replicates was defined as the LOD.

### Application to traditional Chinese medicine herbal piece samples

Six commonly used TCM herbal pieces, including Astragali radix, Angelicae sinensis radix, Glycyrrhizae radix, Codonopsis pilosula radix, Poria cocos, and Glycyrrhiza uralensis radix, were powdered to mimic real-world raw materials. Known concentrations of *P. aeruginosa* were spiked into these samples, and DNA extraction included additional grinding and lysis steps to ensure efficient recovery.

#### Parallel validation using qPCR

To evaluate the diagnostic performance of the LAMP-LFS assay, parallel validation experiments were conducted using quantitative real-time PCR (qPCR) targeting the same *ecfX* gene. A total of 90 TCM herbal piece samples (40 spiked samples with known *P. aeruginosa* concentrations, 20 sterile negative control samples, and 30 naturally contaminated field-collected samples) were tested in parallel by both LAMP-LFS and qPCR. The qPCR reaction was performed in a total volume of 20 μL containing 10 μL of 2× TB Green Premix Ex Taq II (Takara, Japan), 0.4 μM each of forward and reverse primers (*ecfX*-qF: 5′-CGGTGGTGCTGTACCTTCTG-3′; *ecfX*-qR: 5′-GCAGCCGAACTGGAAGATGT-3′), and 2 μL of DNA template. Cycling conditions were as follows: 95 °C for 30 s, followed by 40 cycles of 95 °C for 5 s and 60 °C for 30 s. A sample was considered positive when the Ct value was < 35 with a single-peak melting curve. LAMP-LFS and qPCR results were interpreted independently by two blinded operators.

### Statistical analysis

All experiments were performed in triplicate, and data are presented as mean ± standard deviation (SD). Diagnostic performance of the LAMP-LFS assay was evaluated by calculating sensitivity, specificity, positive predictive value (PPV), negative predictive value (NPV), and accuracy using qPCR as the reference method. Agreement between LAMP-LFS and qPCR results was assessed using Cohen’s kappa coefficient. Statistical analyses were performed using SPSS version 26.0 (IBM Corp., Armonk, NY, USA). The 95% confidence intervals (CIs) for diagnostic performance estimates were calculated using the Clopper–Pearson exact method.

## Results

### Specificity of the *ecfX*-LAMP assay in *P. aeruginosa* and herbal piece samples

**Figure 1:**
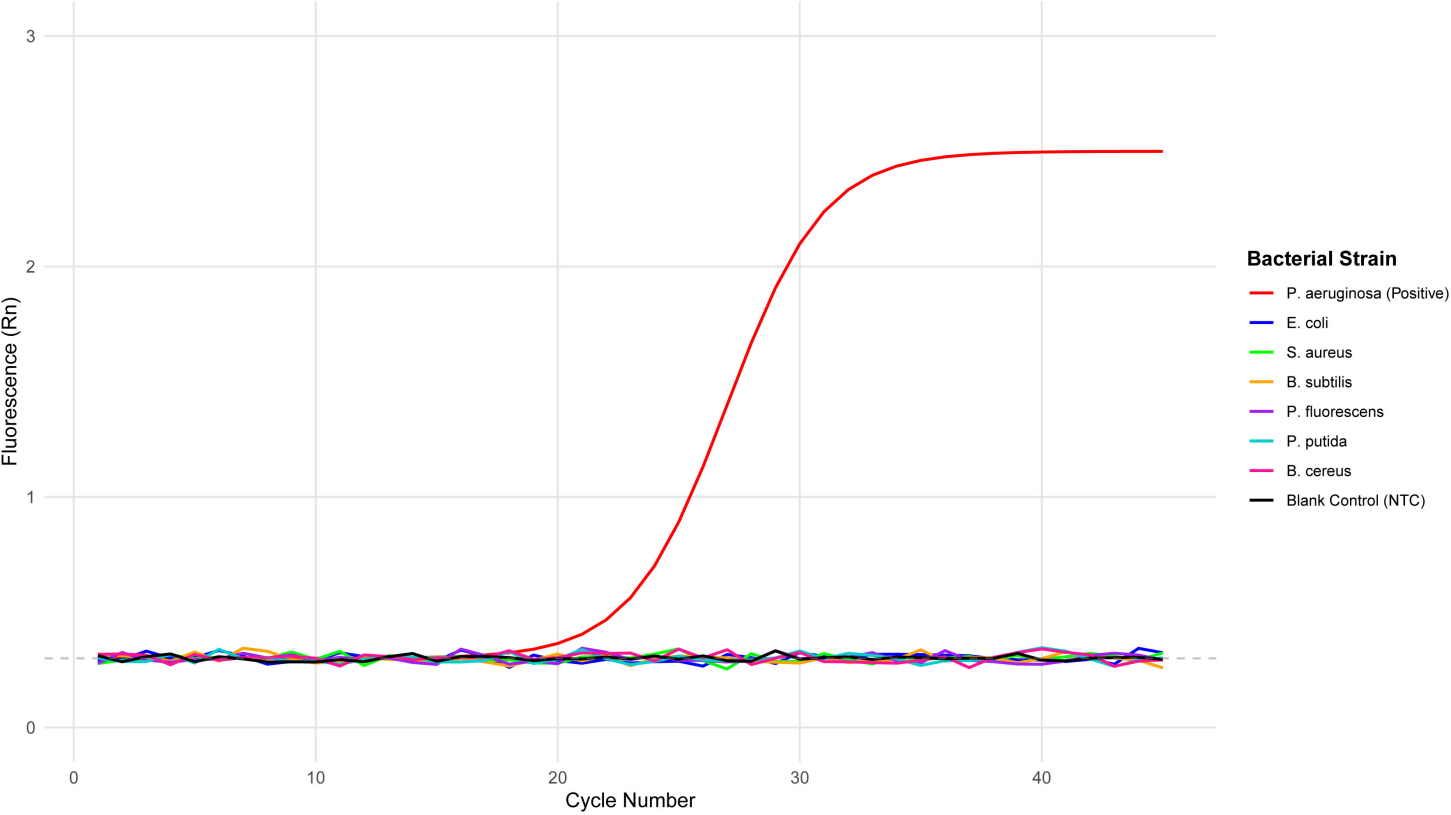
Specificity of the *ecfX*-LAMP assay. Real-time fluorescence amplification curves for *P. aeruginosa* (ATCC 27853) and non-target bacterial strains (*Escherichia coli*, *Staphylococcus aureus*, *Bacillus subtilis, Pseudomonas fluorescens, Pseudomonas putida, Bacillus cereus*).

The specificity of the *ecfX*-LAMP assay was evaluated using genomic DNA from *P. aeruginosa* (ATCC 27853) and several representative non-target bacterial strains, including *Escherichia coli* (ATCC 25922), *Staphylococcus aureus* (ATCC 29213), *Bacillus subtilis* (ATCC 6051), *Pseudomonas fluorescens* (ATCC 13525), *Pseudomonas putida* (ATCC 12633), and *Bacillus cereus* (ATCC 14579). Positive amplification signals were observed exclusively for *P. aeruginosa*, showing the typical fluorescence amplification curve (**Figure 1**). All six non-target strains and the blank control produced no amplification, confirming high assay specificity.

### Optimization of LAMP reaction conditions for herbal piece matrices

**Figure 2:**
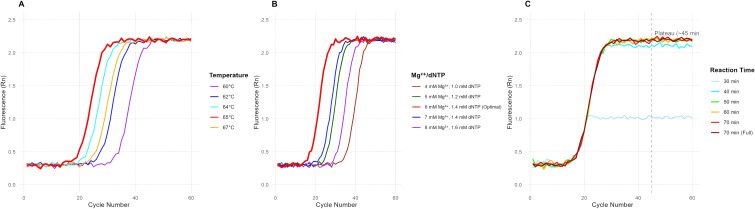
Optimization of LAMP reaction conditions in herbal piece matrices. **(A)** Fluorescence amplification curves under different temperatures (60–67 °C). **(B)** Amplification curves with varying Mg²^+^ and dNTP concentrations. **(C)** Time-course amplification curves showing plateau phase under different reaction times.

LAMP reaction parameters were systematically optimized in spiked herbal piece samples, including temperature (60–67 °C), Mg²^+^ concentration, dNTP concentration, and incubation time (30–70 min). The optimal conditions were determined to be 65 °C, 6 mM Mg²^+^, 1.4 mM dNTPs, and 60 min (**Figure 2**). Under these conditions, fluorescence amplification curves reached plateau within 45 min.

### Characterization of FMs–SA conjugates

**Figure 3:**
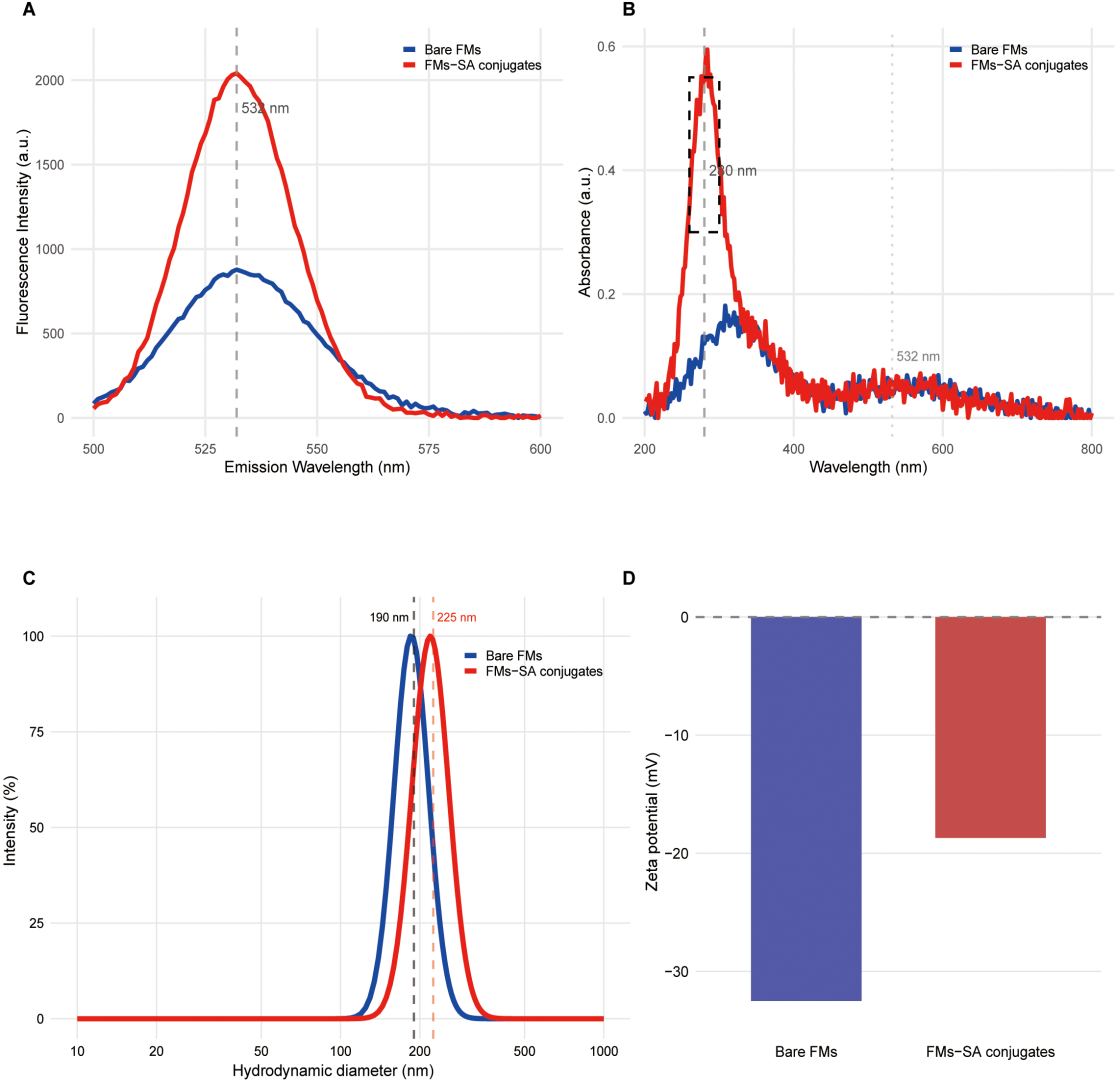
Characterization of fluorescent microsphere–streptavidin (FMs–SA) conjugates. **(A)** Fluorescence emission spectra of bare FMs and FMs–SA conjugates. **(B)** UV–visible absorption spectra of bare FMs and FMs–SA conjugates. **(C)** DLS analysis showing hydrodynamic diameter changes before and after streptavidin conjugation. **(D)** Zeta potential analysis demonstrating surface charge shift after FMs–SA coupling.

Fluorescent microsphere–streptavidin (FMs–SA) conjugates were successfully prepared using EDC/Sulfo-NHS chemistry. Fluorescence spectroscopy confirmed a strong emission peak at 532 nm, indicating successful conjugation, while UV–vis absorption spectra showed the characteristic streptavidin absorption at 280 nm (**Figures 3A, B**). DLS analysis revealed that the Z-average hydrodynamic diameter increased from 183.4 ± 4.2 nm for bare FMs to 218.7 ± 5.6 nm for FMs-SA conjugates, with PDI values of 0.09 ± 0.01 and 0.13 ± 0.02, respectively (**Figure 3C**). Zeta potential measurements showed a shift from −44.2 ± 2.8 mV for bare FMs to −20.3 ± 2.1 mV for FMs-SA conjugates (**Figure 3D**).

### Performance of the lateral flow strip detection system with herbal piece sample

**Figure 4:**
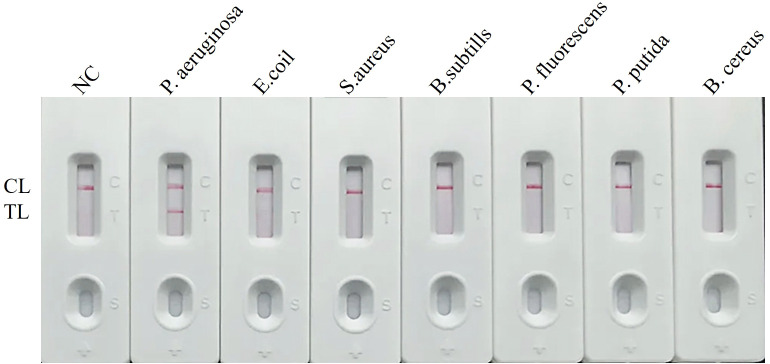
Lateral flow strip detection of LAMP amplicons in spiked herbal piece samples. Representative images of LFS showing T line and C line for *P. aeruginosa* and non-target bacterial DNA.

The LAMP amplicons from spiked herbal samples were applied to LFS, producing clear fluorescent signals at both the T line and C line within 10 min (**Figure 4**). Non-target bacterial DNA generated only the C line, confirming specific detection in complex herbal matrices.

### Optimization of lateral flow strip conditions for herbal piece matrices

**Figure 5:**
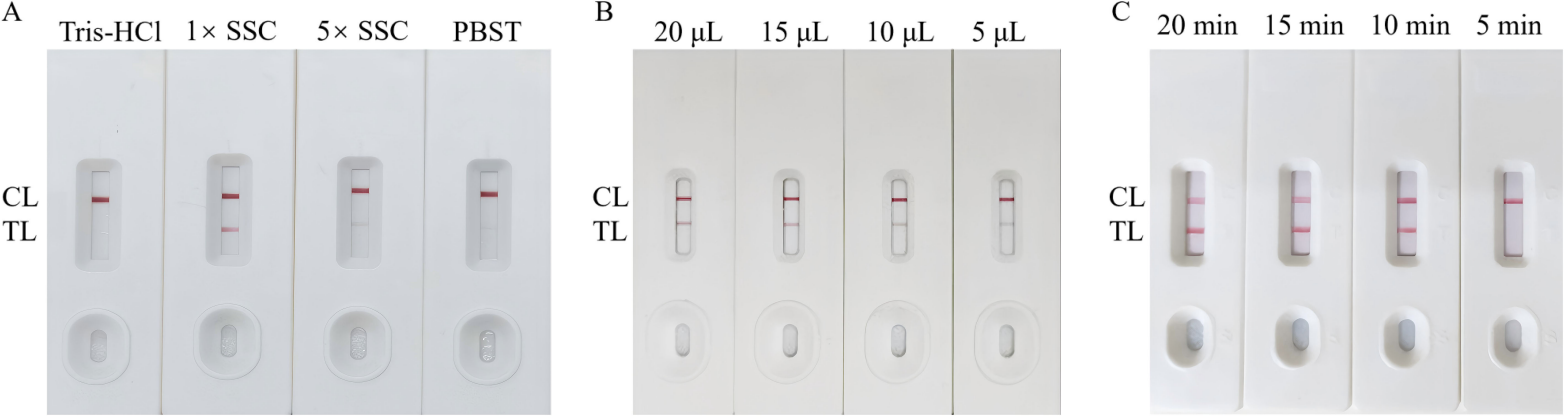
Optimization of lateral flow strip conditions. **(A)** Comparison of running buffer types on strip performance. **(B)** Effect of FMs–SA conjugate loading volume on strip signal. **(C)** Effect of development time on strip signal intensity.

Key parameters affecting LFS performance were systematically optimized, including running buffer type and volume, FMs–SA loading amount, and development time. Among tested buffers, 1× SSC containing 0.5% Tween-20 provided the highest signal-to-noise ratio. The optimal loading volume of FMs–SA was 15 μL per strip, and the ideal strip development time was 10 min (**Figure 5**).

### Sensitivity, specificity, reproducibility, and limit of detection (LOD) in herbal piece matrice

**Figure 6:**
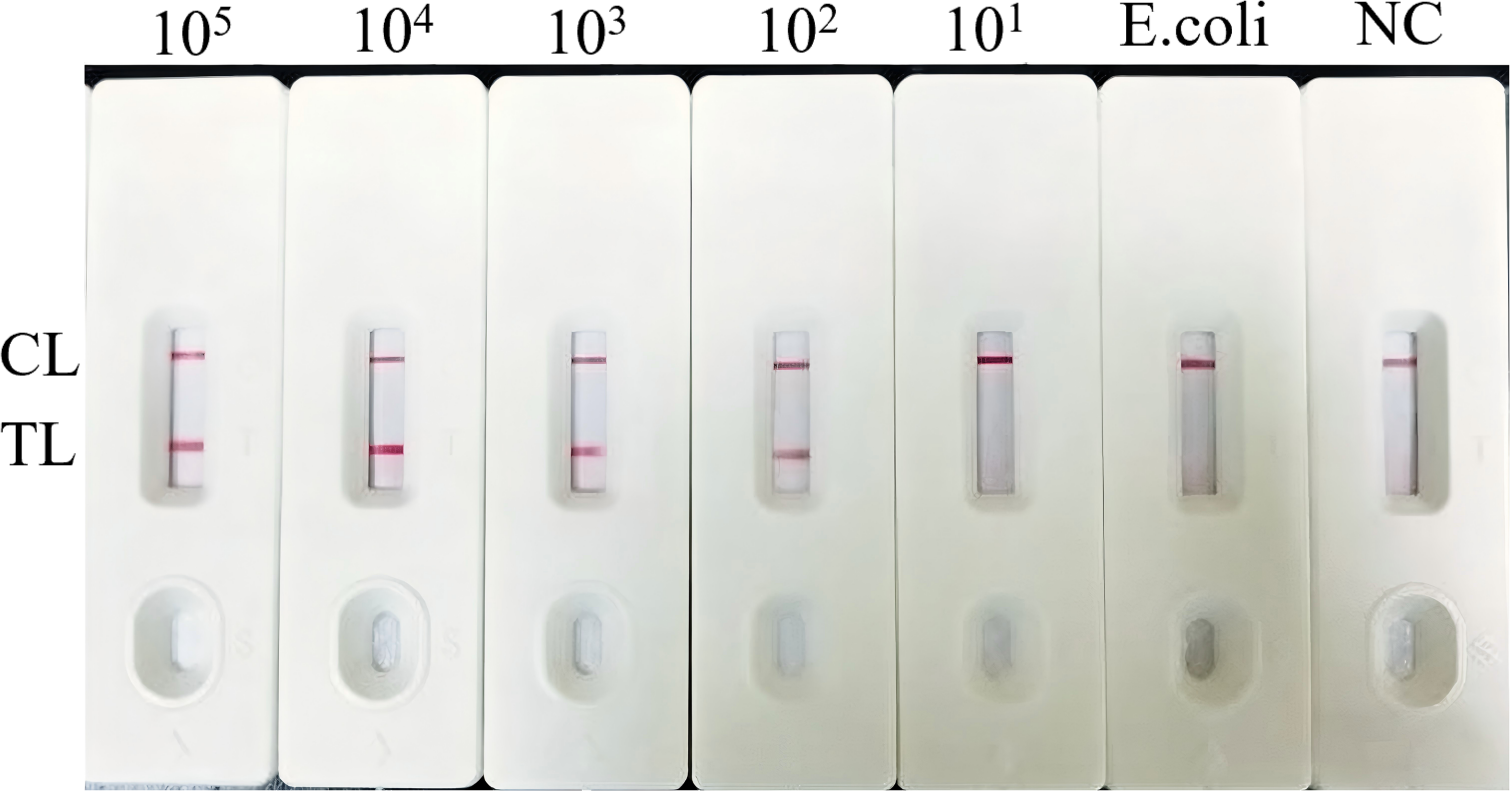
Sensitivity and reproducibility testing of the LAMP-LFS system in spiked herbal piece matrices. LFS images for serial dilutions of *P. aeruginosa ecfX* gene (10^1–^10^5^ CFU/250 mL), showing triplicate measurements for each concentration.

Serial dilutions of *P. aeruginosa ecfX* gene templates (10^1–^10^5^ CFU/250 mL) were spiked into powdered herbal pieces to assess assay sensitivity. The LAMP-LFS system consistently detected as low as 1 × 10² CFU/250 mL, with a CV <5% across three replicates (**Figure 6**). Non-target bacteria produced no false-positive signals, confirming high specificity.

### Application to traditional Chinese medicine herbal pieces

**Figure 7:**
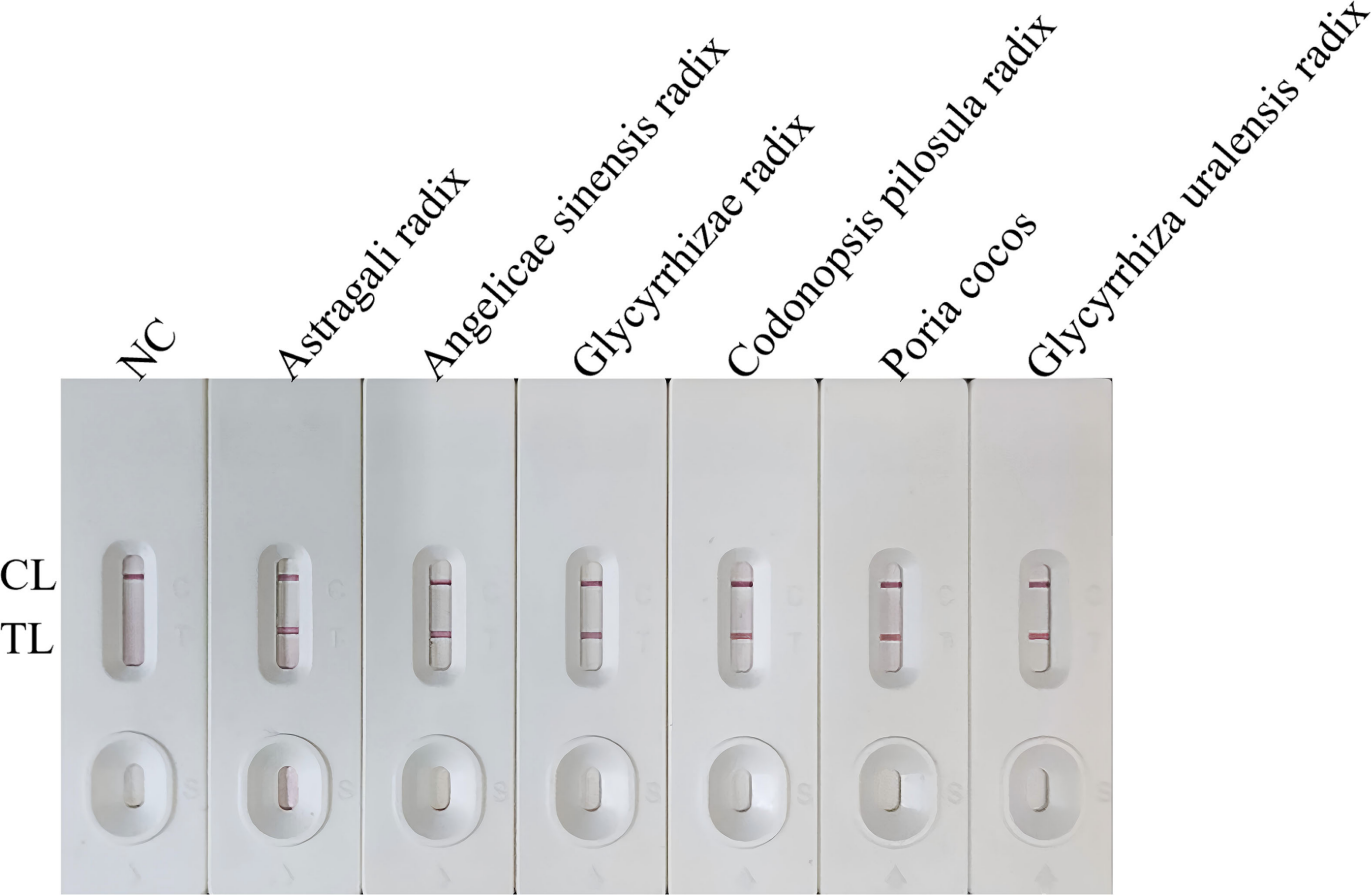
Application of the LAMP-LFS system to six TCM herbal pieces. Representative LFS images for Astragali radix, Angelicae sinensis radix, Glycyrrhizae radix, and three other herbal pieces spiked with serial dilutions of *P. aeruginosa*.

Six commonly used TCM herbal pieces (Astragali radix, Angelicae sinensis radix, Glycyrrhizae radix, etc.) were used to evaluate the performance of the LAMP-LFS assay in complex herbal matrices. The optimized LAMP-LFS assay successfully detected *P. aeruginosa* in all tested herbal samples (**Figure 7**).

### Parallel validation of LAMP-LFS using qPCR

To evaluate the diagnostic performance of the LAMP-LFS assay, parallel validation was conducted using qPCR as the reference method on 90 TCM herbal piece samples. As shown in **Table 2**, the LAMP-LFS assay showed 47 true positives, 39 true negatives, 1 false positive, and 3 false negatives, with 86 out of 90 samples (95.6%).

**Table 2:** Comparison of LAMP-LFS and qPCR for detection of *P. aeruginosa* in TCM herbal pieces (stratified by sample type).

Sample type	N	TP	TN	FP	FN
Spiked samples	40	38	0	0	2
Negative control samples	20	0	20	0	0
Naturally contaminated samples	30	9	19	1	1
Total	90	47	39	1	3

TP, true positive; TN, true negative; FP, false positive; FN, false negative.

The diagnostic performance metrics are summarized in **Table 3**. The LAMP-LFS assay demonstrated a sensitivity of 94.0% (95% CI: 83.5–98.7%), a specificity of 97.5% (95% CI: 86.8–99.9%), a PPV of 97.9% (95% CI: 89.3–99.7%), a NPV of 92.9% (95% CI: 79.9–98.3%), and an overall accuracy of 95.6% (95% CI: 89.1–98.5%). The Cohen’s kappa coefficient was 0.92 (95% CI: 0.85–0.97).

**Table 3:** Diagnostic performance of LAMP-LFS for detection of *P. aeruginosa* using qPCR as the reference method.

Parameter	Value	95% CI
Sensitivity (%)	94.0	83.5–98.7
Specificity (%)	97.5	86.8–99.9
Positive predictive value (PPV, %)	97.9	89.3–99.7
Negative predictive value (NPV, %)	92.9	79.9–98.3
Diagnostic accuracy (%)	95.6	89.1–98.5
Cohen’s kappa coefficient	0.92	0.85–0.97

Calculations are based on total sample size (N = 90). Sensitivity, TP/(TP+FN); Specificity, TN/(TN+FP); PPV, TP/(TP+FP); NPV, TN/(TN+FN); Accuracy, (TP+TN)/N. Kappa coefficient interpretation: 0.81–1.00 indicates almost perfect agreement.

## Discussion

In this study, we developed and systematically evaluated a LAMP assay combined with fluorescent LFS for the rapid detection of *P. aeruginosa* in complex TCM herbal matrices. The assay achieved a LOD of 1 × 10² CFU/250 mL, excellent specificity against non-target bacteria including *Escherichia coli*, *Staphylococcus aureus*, and *Bacillus subtilis*, and high reproducibility (CV <5%) across six representative herbal species. In addition, specificity testing against closely related species, including *Pseudomonas fluorescens* and *Pseudomonas putida*, demonstrated no cross-reactivity, further supporting the species specificity of the *ecfX*-targeted assay. With a total turnaround time of approximately 70 min, including amplification and LFS readout, the developed platform enables rapid detection with minimal equipment requirements, demonstrating its practical utility for routine microbiological quality control of TCM raw materials.

The high sensitivity and specificity achieved herein can be attributed to the synergistic advantages of LAMP and fluorescent LFS. TCM herbal matrices are rich in polysaccharides, polyphenols, pigments, and other secondary metabolites that frequently inhibit thermolabile DNA polymerases in PCR-based assays, resulting in false-negative outcomes (Moon et al., 2022). The Bst 3.0 polymerase employed in LAMP, by contrast, possesses inherent strand-displacement activity and markedly greater tolerance to such inhibitory compounds, enabling robust amplification even in the presence of complex botanical backgrounds (Oscorbin and Filipenko, 2023). Furthermore, dual-labeled primers (digoxigenin and biotin) enable selective capture of target amplicons at the test and control lines of the LFS, thereby minimizing non-specific binding and permitting semi-quantitative result interpretation through fluorescent microsphere signal intensity (Warmt et al., 2022; Agarwal et al., 2023). Several previous studies have reported *ecfX*-targeted LAMP assays or LAMP–LFS platforms for the detection of *P. aeruginosa* in food, environmental, or clinical samples (Lavenir et al., 2007; Yang et al., 2021). However, relatively few investigations have focused on TCM herbal materials, which represent substantially more challenging analytical matrices due to their diverse phytochemical composition and the presence of amplification inhibitors. In the present study, assay development and optimization were performed directly using herbal piece matrices, including optimization of DNA extraction procedures, amplification conditions, and strip operating parameters. This matrix-oriented strategy may have contributed to the stable amplification performance observed across multiple herbal species.

Compared with previously reported LAMP–LFS assays for food or clinical samples, which generally achieve LODs of 10³–10^4^ CFU/mL (Marino et al., 2025), the present method demonstrated superior sensitivity in complex herbal matrices. This improvement is attributable to several factors: (i) primer sequences targeting the highly conserved *ecfX* gene of *P. aeruginosa*; (ii) systematic optimization of reaction conditions, including temperature, Mg²^+^ concentration, and dNTP ratios; and (iii) the incorporation of fluorescent microsphere–streptavidin conjugates for signal amplification on the LFS. While most prior LAMP–LFS studies have focused on relatively homogeneous food or clinical specimens, TCM herbal pieces introduce additional challenges stemming from their heterogeneous phytochemical profiles and variable inhibitor content (Park, 2022; Vajpayee et al., 2023). Notably, characterization of the fluorescent microsphere–streptavidin conjugates by DLS and Zeta potential analysis demonstrated successful surface modification, with an increase in hydrodynamic diameter and a corresponding shift in surface charge following conjugation. These findings support the stability of the signal-reporting system used in the LFS platform and may partially contribute to the consistent strip performance observed during assay optimization.

Conventional culture-based methods for detecting microorganisms in TCM herbal materials typically require 2–7 days and yield LODs of 10³–10^5^ CFU/g, whereas qPCR achieves faster results (2–4h) with LODs of approximately 10²–10³ CFU/g but remains susceptible to matrix-derived inhibition (Wang et al., 2022). In contrast, the present LAMP–LFS assay combines a short turnaround (~70 min) with high sensitivity (LOD 1 × 10² CFU/250 mL) and robust inhibitor tolerance, demonstrating a clear practical advantage for decentralized quality control. Moreover, parallel validation against qPCR using the same batch of herbal samples showed high agreement between the two methods. The assay achieved a sensitivity of 94.0%, specificity of 97.5%, overall accuracy of 95.6%, and a Cohen’s kappa coefficient of 0.92. Only one false-positive and three false-negative results were observed among the 90 tested samples. These findings suggest that the developed assay provides reliable detection performance under practical testing conditions while maintaining operational simplicity. By systematically optimizing both LAMP and LFS parameters for these demanding botanical matrices, this work addresses a critical methodological gap in rapid microbial detection for herbal products.

The practical implications of this assay merit emphasis. Timely and reliable detection of *P. aeruginosa* is an essential component of microbiological safety assurance for TCM herbal pieces, which remain in widespread clinical use. The method’s short turnaround time, minimal equipment requirements, and semi-quantitative readout capability collectively render it well-suited for on-site monitoring at TCM manufacturing facilities, distribution warehouses, hospital pharmacies, and regulatory inspection points. Because amplification is performed under isothermal conditions and result interpretation relies on a portable LFS format, the assay may be particularly suitable for settings where access to conventional molecular diagnostic equipment is limited. Integration of this platform into existing quality control workflows could substantially reduce contamination risk, accelerate batch-release decisions, and strengthen compliance with pharmacopoeial microbial limit standards for herbal medicines.

Despite these promising results, several limitations warrant acknowledgment. First, the assay was validated on six representative herbal species; its performance in herbs with exceptionally high concentrations of tannins, saponins, or other potent inhibitors may require additional evaluation. Second, the current format targets only P. aeruginosa, restricting its utility for panels of microbial contaminants commonly monitored in TCM quality control. Third, the absence of internal amplification controls precludes discrimination between true negatives and inhibition-related false negatives. Fourth, the DNA extraction protocol, although optimized for herbal matrices, requires approximately 30 min and multiple manual steps, which may limit throughput in high-volume settings. Finally, although high agreement with qPCR was demonstrated, the number of naturally contaminated field samples remained relatively limited, and larger validation studies involving broader geographic regions and herbal species would further strengthen the evidence supporting routine implementation.

Future work should address these limitations through: (i) expansion of the validation panel to encompass a broader range of TCM herbal species, including those with known high inhibitor content; (ii) development of multiplex LAMP–LFS formats using differentially labeled primer sets and multiple test lines for simultaneous pathogen detection; (iii) incorporation of non-competitive internal controls (e.g., exogenous DNA templates) to monitor extraction efficiency and amplification integrity; (iv) streamlining of DNA extraction through magnetic bead–based or direct lysis approaches compatible with field deployment; and (v) integration with portable fluorescence readers and smartphone-based image analysis to enable quantitative, high-throughput on-site testing. Such developments would facilitate broader regulatory acceptance and adoption of LAMP–LFS platforms within the TCM industry.

In conclusion, the fluorescent microsphere–based LAMP–LFS assay developed in this study provides a rapid, sensitive, specific, and reproducible method for detecting *P. aeruginosa* in TCM herbal pieces. Unlike most previously reported *ecfX*-targeted LAMP or LAMP–LFS assays that were evaluated in food, environmental, or clinical samples, the present study demonstrates the applicability of this approach in complex herbal matrices. The assay may serve as a useful tool for routine quality control and on-site microbial monitoring of TCM raw materials.

## Data Availability

The raw data supporting the conclusions of this article will be made available by the authors, without undue reservation.
